# An Unusual Presentation of Scimitar Syndrome in a Military Service Member

**DOI:** 10.1155/2013/632402

**Published:** 2013-07-24

**Authors:** Daniel B. Simmons, Ravi S. Menon, William L. Pomeroy, Travis C. Batts, Ahmad M. Slim

**Affiliations:** ^1^Cardiology Service, Brooke Army Medical Center, San Antonio, TX 78234, USA; ^2^Cardiology Service, San Antonio Army Medical Center, MCHE-MDC, 3851 Roger Brooke Drive, San Antonio, TX 78234-6200, USA

## Abstract

This is the case of a twenty-two-year-old active duty male soldier with nonexertional chest pain and worsening performance on his physical fitness test. His history was significant for a diagnosis of dextrocardia upon entry to the military. On acute presentation to the emergency department, he was deemed a candidate for the expedited coronary computed tomographic angiography (CCTA) protocol to assess for a possible anatomic cause of his symptoms. CCTA revealed the presence of an anomalous right pulmonary vein draining into the inferior vena cava. Additionally, the imaging showed dextroversion of the heart, dilation of the inferior vena cava, right atrium, and right ventricle, as well as a hypoplastic right lung, a collection of findings consistent with scimitar syndrome and not dextrocardia.

## 1. Case Report

This is a case report of a twenty-two-year-old male soldier with nonexertional chest pain and worsening performance on his physical fitness test. His history was significant for a diagnosis of dextrocardia upon entry to the military. On physical examination, heart sounds were louder over the right hemithorax; there was a fixed split S2, and the point of maximal impulse was displaced to the right. He had normal lung sounds and no peripheral edema. An electrocardiogram was remarkable for left axis deviation of the p-wave, normal QRS axis, and a nonspecific intraventricular conduction delay. 

**Figure 1:**
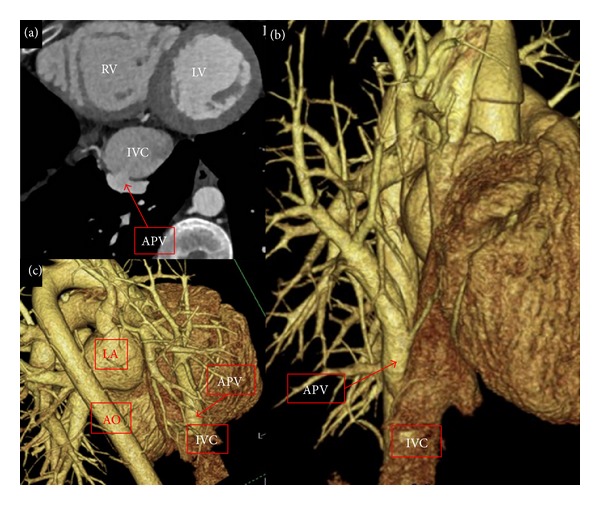
Axial and coronal images with sequential gating; cardiac phases utilized of 40–80% on CCTA (kVp = 120 with care dose variable mAs and slice thickness of 0.7 mm with 0.4 overlap). They revealed the presence of an anomalous pulmonary venous connection between the right lower lobe pulmonary vein (APV) and the enlarged inferior vena cava (IVC) [a, b, and c].

Because of his history of recurrent chest pain and vague history of dextrocardia not confirmed with ECG, patient underwent a cardiac computed tomographic angiography (CCTA) protocol to assess for a possible anatomic cause of his symptoms. CCTA, performed with sequential gating and a window of 40–80% to accommodate the large area under investigation, revealed the presence of an anomalous right pulmonary vein draining into the inferior vena cava. Additionally, the imaging showed dextroversion of the heart, dilation of the inferior vena cava, right atrium, and right ventricle, as well as a hypoplastic right lung, a collection of findings consistent with scimitar syndrome and not dextrocardia (Figure 1). There was no evidence of pulmonary sequestration or an atrial septal defect.

**Figure 2:**
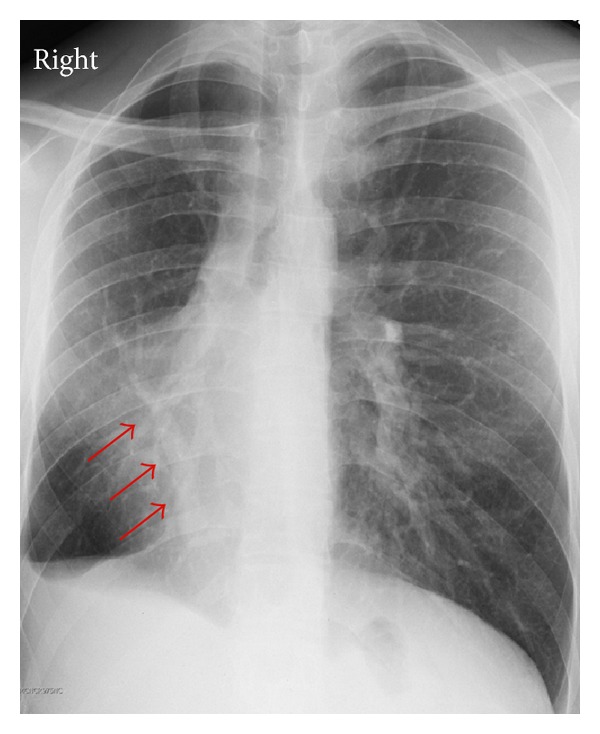
Chest radiograph shows decreased space associated with the hypoplastic lung and displacement of the cardiac silhouette to the right (scimitar sign) (arrows).

**Figure 3:**
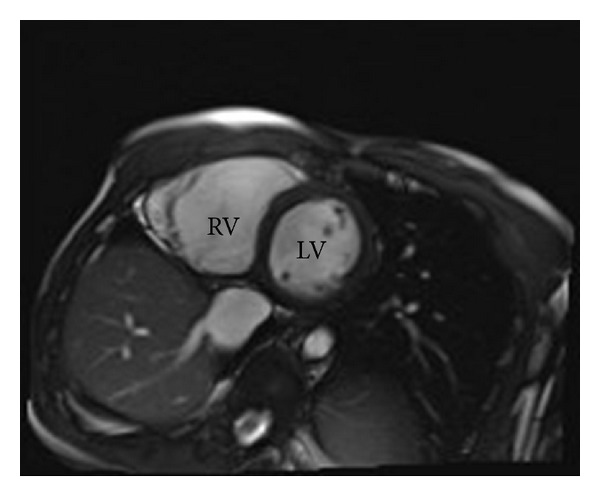
Cardiac magnetic resonance imaging showing right ventricular (RV) enlargement as compared to the left ventricular (LV) size with calculated right ventricular ejection fraction of 35%.

Review of the patient's chest radiograph showed a classic scimitar sign, which is the presence of a scimitar-shaped pulmonary vein along the right cardiac border (Figure 2). Due to concerns about RV enlargement, cardiac magnetic resonance imaging was performed and confirmed the finding of right ventricular enlargement on CCTA and estimated the right ventricular ejection fraction at 35% (Figure 3). Catheterization of the right heart revealed a step-up in oxygenation of the upper inferior vena cava consistent with a left-to-right shunt, normal right-sided pressures without evidence of pulmonary hypertension, and a pulmonary to systemic shunt fraction (Qp/Qs) of 1.63. 

## 2. Discussion

Scimitar syndrome is a rare form of anomalous pulmonary venous return with an estimated incidence of 2/100,000 in which all or part of the venous drainage from the right lung enters the inferior vena cava instead of the left atrium [1]. The syndrome derives its name from the classical appearance of the chest radiograph, in which the shape of the anomalous pulmonary vein resembles the curved Turkish sword [2]. The severity of symptoms depends on the degree of the shunt present and may be manifested as dyspnea on exertion, palpitations, syncope, or congestive heart failure [3]. Pulmonary sequestration with systemic arterial supply and atrial septal defect is present in approximately 50% and 20% of adults, respectively [1]. There are three variants of scimitar syndrome: an infantile form which is diagnosed before one year and is associated with heart failure and pulmonary hypertension, an adult form with milder symptoms, and a form associated with other congenital heart malformations [4].

Scimitar syndrome has classically been diagnosed in the cardiac catheterization laboratory, but CCTA—as part of a routine screening protocol in our institution for low-risk chest pain—was essential to make the diagnosis. It also demonstrated the right-sided pulmonary hypoplasia and dextroversion of the heart which are the most common abnormalities associated with scimitar syndrome. The patient ultimately proceeded to surgical correction of the anomalous pulmonary venous return, and CCTA was vital in operative planning and successful surgical repair. 

## 3. Conclusion

Scimitar syndrome is a rare variant of anomalous pulmonary venous return that is usually diagnosed in infanc, but may present in adults with symptoms of dyspnea on exertion, syncope, or heart failure. In this twenty-two-year-old soldier with prior combat deployments, CCTA was vital in the diagnosis of scimitar syndrome. This vascular anomaly and the associated congenital malformations likely led to the soldier's decreased exercise tolerance and were successfully repaired by a congenital heart specialist.
